# Leadership and management competencies and practices among health managers in Wakiso District, Uganda

**DOI:** 10.1371/journal.pone.0356074

**Published:** 2026-08-14

**Authors:** David Musoke, Allan Ssembuusi, Grace Biyinzika Lubega, Filimin Niyongabo, Michael Obeng Brown, Richard Holder, Russell Pitchford, Linda Gibson

**Affiliations:** 1 Department of Disease Control and Environmental Health, Makerere University School of Public Health, Kampala, Uganda; 2 Institute of Health and Allied Professions, School of Social Sciences, Nottingham Trent University, Nottingham, United Kingdom; 3 Nottingham University Hospitals, NHS Trust, Nottingham, United Kingdom; World Health Organization, CONGO

## Abstract

**Introduction:**

Leadership and management are of great importance for health service delivery, yet they are understudied in Uganda. We therefore conducted a study that assessed the leadership and management competencies and practices among health managers in Wakiso District, Uganda.

**Materials and Methods:**

A cross-sectional study that employed a structured questionnaire was conducted among 60 health managers. The health managers were in-charges of government lower-level health facilities (health centre II, III, and IV). The questionnaire had questions on leadership and management competencies including cognitive, interpersonal, organisation and supervision, as well as human resource management skills. Data was analysed descriptively in Stata version 14.

**Results:**

Most health managers were female 37 (61.7%), a significant proportion 43 (71.7%) were not inducted into their roles, and 37 (61.7%) lacked motivation to perform their duties. Overall, only 15 (25.0%) and 20 (33.3%) demonstrated good leadership and management competencies respectively. In addition, means for leadership and management competencies ranged from 20.9 to 37.0 and 7.8 to 36.5 respectively. According to the health managers, monitoring 39 (65.0%), speaking 36 (60.0%) and active listening 35 (58.3%) were the most important cognitive skills; while coordination 27 (45.0%), instructing 25 (41.7%), and social perceptiveness 25 (41.7%) were mentioned as the most important interpersonal skills. In addition, management of financial 38 (63.3%), logistic 34 (56.7%), and human resources 31 (52.5%) were the most important business analysis skills. Health managers frequently engaged in promoting good working relationships among subordinates 32 (53.3%), holding self and others accountable for health facility organisational goal attainment 28 (46.7%), and creating an effective work environment for subordinates 27 (45.0%).

**Conclusions:**

Leadership and management competencies among health managers were generally lacking. There is need for induction and routine refresher training on leadership and management among health managers to enhance their performance.

## Introduction

Leadership and management competencies are recognised globally as fundamental for effective healthcare delivery [1–4]. A good leader is one who can select, equip, train, influence and direct followers toward achieving the organisation’s mission and objectives [5]. Similarly, a good manager is one who can organise, coordinate and direct resources such as personnel, facilities, and finances to efficiently and effectively deliver services [6,7]. Therefore, a competent manager is one who has the technical skills, knowledge and attitudes required to perform a job [8]. In the context of leadership and management for health facilities, health managers’ competencies extend to overseeing all healthcare operations including strategic planning, organising, staffing, directing, and implementation of quality improvement initiatives for better healthcare outcomes within the available resources [9,10]. For many years, the World Health Organization (WHO) has reported weaknesses in management and leadership competencies among health managers in lower-tier health systems [6,11]. These incompetencies have mainly been linked to poor healthcare service delivery, misallocation and wastage of resources, low staff morale towards work, loss of trust by the community, poor health indicators, as well as compromised healthcare outcomes [12,13]. Many low- and middle-income countries (LMICs), especially those in sub-Saharan Africa, have a shortage of purpose-trained health managers [14,15]. Therefore, a number of them assume managerial roles with limited or without specific formal management training [7,16].

In Uganda, the ever-evolving nature of the healthcare sector demands for leaders and managers with a vision, dedication, inspiration, and strong change management skills. However, many health managers in the country lack leadership and management capabilities [15,17]. This is further exacerbated by poor facilitation of human resources for health, inadequate infrastructure and supplies, and poor working environments in the country [18]. Underdeveloped leadership and management in health facilities can lead to significant adverse effects on various aspects of the facility operations. These effects may include compromised quality of patient care, decreased staff morale and retention rates, financial losses due to ineffective financial management, inefficient resource utilisation, prolonged patient wait times, and increased legal and regulatory risks [19]. In addition, these challenges can collectively undermine the health facility’s reputation within the community and among other stakeholders, ultimately impacting its ability to effectively deliver high-quality healthcare services [13]. However, literature suggests that competent health managers can ensure provision of quality services, optimisation of resource utilisation, and can easily address dynamic challenges at health facilities [20]. In addition, WHO argues that health managers with good leadership principles are able to guide vision, while those with sound management practices can ensure efficient utilisation of resources for healthcare institutions [7].

Uganda has over 6,900 health facilities across the country, with a total of 3,134 (45%) being government owned. These include national and regional referral hospitals, general hospitals and lower-level health facilities. Lower-level health facilities are governed by local governments at districts and are graded as health centre (HC) IIs at parish or ward level, HC IIIs at sub-county or division level, and HC IVs at county or municipal level. HC IIs are usually headed by nurses and provide preventive, promotive and outpatient curative health services, outreach and emergency core to a population of approximately 5,000 people. HC IIIs are usually headed by clinical officers and serve an estimated population of 20,000 people providing all HC IIs services in addition to maternity, inpatient and laboratory services. HC IVs are headed by a medical officer and serve an estimated population of 100,000 people offering all HC III services in addition to emergency surgery and blood transfusion services [21]. Despite the acknowledged significance of healthcare leadership and management in health service delivery [22–24], there is still a critical gap in understanding the capabilities of lower-level health managers. Existing literature on leadership and management primarily focuses on high-level administrators such as district and hospital managers [1,2,24,25]. Therefore, limited research has been carried out among lower-level health managers, despite them providing health services to most of the population. This study therefore assessed the leadership and management competencies and practices among lower-level health managers in Wakiso District, Uganda.

## Methods

### Study area and context

The study was conducted among managers who were health facility in-charges in Wakiso District, Uganda. Wakiso is situated in Uganda’s central region and partially surrounds Kampala, the country’s capital city. The district constitutes of 4 municipalities of Entebbe, Nansana, Makindye Ssaabagabo and Kira which is the second-largest municipality in Uganda. Wakiso is bordered by Nakaseke and Luweero districts in the North, Mukono district to the East, Kalangala district to the South, Mpigi district to the Southwest, and Mityana district to the Northwest. As of the 2024 census, the district’s population was 3,397,555 [26]. The district is divided into two counties – Kyadondo and Busiro. These counties are further divided into 8 constituencies, 8 sub-counties, 8 town councils, 148 parishes, and 704 villages. Wakiso district was particularly chosen for this study because of a planned project to enhance leadership and management of local government health managers in the area. This project was to be implemented as part of the partnership between Nottingham Trent University and Makerere University in collaboration with Nottingham University Hospitals NHS Trust and the Uganda Ministry of Health. The district has 234 clinics, 153 HC IIs, 165 HC IIIs, and 19 HC IVs, with a regional referral hospital (Entebbe Regional Referral Hospital) [21]. Among these health facilities, only 72 are government owned. Among the government owned facilities, 60 are directly overseen by Wakiso District Health Office, and 12 are under the oversight of other parastatals such as the State House, Uganda Prisons and other security agencies [21].

### Study design and sampling

This cross-sectional study was conducted among managers of all the health facilities that were directly supervised by Wakiso District Local Government. A structured questionnaire was used to collect data between 27^th^ May to 21^st^ June 2024 on the level of leadership and management competencies and practices at the time of the study. All the 60 managers of health facilities were involved as follows: 27 HC IIs, 26 HC IIIs and 7 HC IVs. Only one health manager per facility participated in the study, and in the absence of a facility in-charge, the assistant was involved.

### Data collection

A total of 4 experienced Research Assistants (RAs) with at least a bachelor’s degree were trained and oriented for 2 days by the researchers to ensure that they were well versed with the tool before data collection. Efforts were made during the training to ensure that the RAs understood the study objectives and methodology. Supervision of the data collection process was done by the researchers to ensure that all information was accurately collected. The questionnaire contained 4 sections: section 1 on socio-demographic (gender, age, marital status, education level, and professional qualification) and background characteristics (level of health centre, duration of in-charge role, number of health workers at the facility, orientation into role, past trainings, and belief of having leadership and management competencies); section 2 on leadership competencies; section 3 on management competencies; and section 4 on leadership and management practices. Leadership competencies assessed were cognitive (speaking, active listening, critical thinking, active learning, writing, reading comprehension, and monitoring), interpersonal (social perceptiveness, empathy, coordination, persuasion, instructing, and cross-cultural flexibility) and business analysis skills (operations analysis, management of human resources, management of financial resources, and management of material/ logistic resources). Management competencies assessed were self-management (time management, effective meetings, communication skills, presentation skills, feedback, negotiation skills, computer literacy, teamwork, and attention to detail), planning and assessment (strategic management, change management, problem identification and solving, planning skills, and project management), organisation and supervision (principles of management, principles of supervision, and ethical leadership), human resource management (functions of personnel management, performance management, leadership and team development), financial management (cost analysis, budgeting, and accountability), information technology management and decision-making (principles of information management, data collection and analysis, principles of decision making and resource allocation, and knowledge of IT application software systems), quality management, monitoring and evaluation (quality management, basic concepts of monitoring, and basic concepts of evaluation), data management (data management planning, use of data for decision making, record keeping, and report writing and submission), and logistics and supply chain management (basic concepts of supply chain management, predict and quantify medicines, and health supplies requirements). The questionnaire was developed with reference to existing literature from previous research [27–31]. This questionnaire was pre-tested among managers of health facilities in Kampala city that neighbours Wakiso district which ensured that the questions were aligned to our research objectives. Reference to leadership and management literature, as well as pretesting the tool enhanced its validity and reliability. Data was collected at the respective facilities at a time most convenient for the participant at the respective health facility. The questionnaire was administered in English on Kobo Collect Application hosted on phone tablets. Real-time checks during data collection were carried out to identify errors or inconsistences.

### Data management and analysis

The data collected was downloaded from the web-based Kobo Collect software, exported to MS Excel 2019 for cleaning, before being transferred to Stata version 14 for analysis. Descriptive analyses were performed using frequencies and percentages for categorical variables, while numerical variables were summarised using means and standard deviations. Leadership competency was assessed with a 17-item tool covering three dimensions of cognitive, interpersonal and business skills. Each item was rated on a seven-point Likert scale (1 = low skill level, 7 = high skill level). Composite scores for each dimension were computed by averaging the respective items, with results presented as means and standard deviations, showing the overall performance in each leadership dimension. In addition, the perceived importance of each leadership skill was assessed separately using a five-point Likert scale (1 = not important, 5 = extremely important), with frequency distributions and percentages presented for each skill. Management competencies were assessed using a 36-item tool organised into 9 dimensions. Each competency was rated on a five-point Likert scale where 1 indicated the manager was not familiar with the competency and had not used it, while 5 indicated the manager had used the competency and could teach it to others. Composite scores for each management dimension were computed as the mean of the respective items and presented alongside standard deviations, reflecting the overall level of expertise among health facility managers. To assess overall leadership competency, total scores across the 17 items were calculated, with the maximum total expected to be 119. Similarly, for overall management competency, total scores across the 36 items were calculated, with the expected maximum total of 180. We obtained 85.7% (102 and above out of 119) and 80% (144 and above out of 180) as cutoffs for overall leadership and management competencies respectively. To obtain the cutoff for leadership competency, the seven-point Likert scale was converted into percentage (1 = 14.3%, 2 = 28.6%, 3 = 42.9%, 4 = 57.1%, 5 = 71.4%, 6 = 85.7%, and 7 = 100%) with scores of ≥6.0 (equivalent to 85.7% or higher) indicating sufficient leadership competency. Similarly, for the management cutoff, the five-point Likert scale was converted into percentage (1 = 20%, 2 = 40%, 3 = 60%, 4 = 80%, and 5 = 100%), with scores ≥4.0 (equivalent to 80% or higher) meeting the management competency cutoff. These cutoffs were seconded after consultations with leadership and management specialists from the Uganda Ministry of Public Service as has been used previously [32] and consensus among the research team. To determine how frequently health managers engaged in leadership and management activities during the week preceding data collection, a five-point Likert scale (1 = never, 5 = always) was used. The frequencies and percentages of responses were computed and presented for each activity.

### Ethics approval and consent to participate

Ethical approval to conduct the study was obtained from Makerere University School of Public Health Research and Ethics Committee (SPH-2024–561). The research was also approved and registered at the Uganda National Council for Science and Technology (HS4164ES). Participation in the study was voluntary, and participants provided written informed consent after explaining to them the proposed research including the anticipated risks and potential benefits before taking part. Before beginning data collection, we obtained administrative permission from the Wakiso District Local Government administration. All data emanating from the study was confidentially stored, with access restricted to only members of the research team.

### Inclusivity in global research

Additional information regarding the ethical, cultural, and scientific considerations specific to inclusivity in global research is included in the Supporting Information (S2 Checklist).

## Results

### Demographic characteristics

Among the 60 health managers, majority 37 (61.7%) were female, with an average age of 42.4 (SD = 7.1) years. Most health managers 27 (45.8%) were diploma holders, and 27 (45.0%) were nursing officers by profession. The average number of years spent as a health manager was 4 (SD = 2.9), with 38 (63.3%) having served for less than five years. Only 17 (28.3%) and (23) 38.3% were inducted and motivated to perform their duties as health managers respectively (Table 1).

**Table 1:** Demographic characteristics of participants.

Variable	Frequency (n = 60)	Percentage (%)
**Position at the health facility**		
In-charge	57	95.0
Assistant in-charge	3	5.0
**Gender**		
Female	37	61.7
Male	23	38.3
**Age (years)**		
30-39	26	43.3
40-49	23	38.3
≥50	11	18.3
Mean/SD (42.4/7.1)		
**Had a disability**		
Yes	2	3.3
No	58	96.7
**Marital status**		
Married/ cohabiting	53	88.3
Single	7	11.7
**Highest level of education**		
Masters	5	8.3
Post-graduate diploma	3	5.0
Bachelors	14	23.3
Diploma	27	45.0
Certificate	11	18.3
**Cadre**		
Clinical officer	23	38.3
Medical officer	5	8.3
Midwife	5	8.3
Nursing officer	27	45.0
**Health facility level**		
Health centre II	27	45.0
Health centre III	26	43.3
Health centre IV	7	11.7
**Number of formal staff at the health facility**		
<10	25	41.7
10-19	24	40.0
20-29	3	5.0
30-39	3	5.0
>39	5	8.3
Mean/SD (16.5/16.1)		
Min/Max (4/80)		
**Duration spent as the health facility in-charge (years)**		
≤4	38	63.3
5-9	17	28.3
≥10	5	8.3
Mean/SD (4.0/2.9)		
Min/Max (1/11)		
**Motivation to perform duties as the health facility in-charge**		
Motivated	23	38.3
Not motivated	37	61.7
**Confidence to perform duties as the health facility in-charge**		
Confident	47	78.3
Not confident	13	21.7
**Conversant with roles of a health facility in-charge**		
Yes	44	73.3
No	16	26.7
**Inducted for the role of the health facility in-charge**		
Yes	17	28.3
No	43	71.7
**Duration of the induction (days) (n = 17)**		
<5	14	82.4
≥5	3	17.7
**Other trainings (apart from the induction) for the role of a health facility in-charge**		
Yes	28	46.7
No	32	53.3
**Time from the last training for the role of a health facility in-charge (years) (n = 28)**		
1	15	53.6
>1	13	46.4
Min/Max (1/15)		
**Duration of the last training for the role of a health facility in-charge (days) (n = 28)**		
1-3	21	75.0
>3	7	25.0
Min/Max (1/7)		
**Belief of having the desired leadership capacity to perform duties as a health facility in-charge**		
Yes	34	56.7
No	26	43.3
**Belief of having the desired management capacity to perform duties as a health facility in-charge**		
Yes	33	55.0
No	27	45.0

### Leadership and management competencies

Less than half 15 (25.0%) of the health managers met the predefined threshold of the 17-item tool assessing leadership competencies, while only 20 (33.3%) met the predefined threshold of the 36-item tool assessing management competencies. Regarding composite scores for leadership and management competencies per dimension, means for leadership competencies ranged from 20.9 to 37.0, with minimums from 12 to 21 and maximums ranging from 27 to 48. For management competencies, their means ranged from 7.8 to 36.5 with minimums and maximums ranging from 3 to 28 and 10–45 respectively. Cognitive skills (37.0) and self-management domains (36.5) were the most prominent under leadership and management competencies respectively (Table 2).

**Table 2:** Composite scores for leadership and management competencies per domain.

Variable	Mean	Standard deviation	Min	Max
**Leadership competencies**				
Cognitive skills	37.0	6.9	20	48
Interpersonal skills	31.6	5.4	21	41
Business analysis skills	20.9	4.1	12	27
**Management competencies**				
Self-management	36.5	4.0	28	45
Planning and assessment	19.8	2.6	13	25
Organisation and supervision	11.3	2.7	3	15
Human resource management	11.6	2.1	3	15
Financial management	12.0	1.7	7	15
Information technology management and decision making	14.5	2.7	8	20
Quality management, monitoring and evaluation	11.1	1.9	5	15
Data management	15.6	2.2	10	20
Logistics and supply chain management	7.8	1.4	4	10

### Importance of leadership competencies

According to the health managers, monitoring 39 (65.0%), speaking 36 (60.0%) and active listening 35 (58.3%) were the three extremely important cognitive skills. For interpersonal skills, coordination 27 (45.0%), instructing 25 (41.7%), and social perceptiveness 25 (41.7%) were mentioned as the three extremely important skills. While management of financial resources 38 (63.3%), management of logistic resources 34 (56.7%), and management of personnel resources 31 (52.5%) were the three extremely important business analysis skills (Table 3).

**Table 3:** Importance of leadership competencies.

Variable	Somewhat important	Important	Very important	Extremely important
	**n (%)**	**n (%)**	**n (%)**	**n (%)**
**Cognitive skills**				
Speaking	0 (0.0)	7 (11.7)	17 (28.3)	36 (60.0)
Active listening	0 (0.0)	4 (6.7)	21 (35.0)	35 (58.3)
Critical thinking	0 (0.0)	5 (8.3)	27 (45.0)	28 (46.7)
Active learning	0 (0.0)	3 (5.0)	26 (43.3)	31 (51.7)
Writing	0 (0.0)	5 (8.3)	29 (48.3)	26 (43.3)
Reading comprehension	0 (0.0)	7 (11.7)	21 (35.0)	32 (53.3)
Monitoring	0 (0.0)	3 (5.0)	18 (30.0)	39 (65.0)
**Interpersonal skills**				
Social perceptiveness	2 (3.3)	6 (10.0)	27 (45.0)	25 (41.7)
Empathy	1 (1.7)	9 (15.0)	29 (48.3)	21 (35.0)
Coordination	0 (0.0)	5 (8.3)	28 (46.7)	27 (45.0)
Persuasion	2 (3.3)	9 (15.0)	31 (51.7)	18 (30.0)
Instructing	0 (0.0)	3 (5.0)	32 (53.3)	25 (41.7)
Cross-cultural flexibility	1 (1.7)	11 (18.3)	29 (48.3)	19 (31.7)
**Business analysis skills**				
Operations analysis	1 (1.7)	6 (10.0)	25 (41.7)	28 (46.7)
Management of personnel resources	0 (0.0)	5 (8.5)	23 (39.0)	31 (52.5)
Management of financial resources	0 (0.0)	2 (3.3)	20 (33.3)	38 (63.3)
Management of material/ logistic resources	0 (0.0)	2 (3.3)	24 (40.0)	34 (56.7)

### Leadership and management practices

Health managers were asked about their engagement in leadership and management practices in the week preceding data collection. Promoting good working relationships among subordinates 32 (53.3%), holding self and others accountable for health facility organisational goal attainment 28 (46.7%), and creating effective work environments for subordinates 27 (45.0%) were the three most practiced activities. However, advocating and participating in healthcare policy initiatives 8 (13.3%), facilitating conflict and alternative dispute resolution among members 12 (20.0%), and supporting and mentoring high-potential talent within the health facility 12 (20.0%) were the three least practiced activities by the health managers (Table 4).

**Table 4:** Leadership and management practices.

Variable	Never	Rarely	Often	Usually	Always
	**n (%)**	**n (%)**	**n (%)**	**n (%)**	**n (%)**
Offered support supervision to their subordinates	2 (3.33)	3 (5.0)	17 (28.3)	16 (26.7)	22 (36.7)
Created an effective work environment for their subordinates	0 (0.0)	1 (1.7)	7 (11.7)	25 (41.7)	27 (45.0)
Prompted participation of subordinates in health facility meetings	5 (8.3)	3 (5.0)	8 (13.3)	24 (40.0)	20 (33.3)
Solicited their subordinates’ input in the day-to-day activities of the health facility	1 (1.7)	1 (1.7)	9 (15.0)	30 (50.0)	19 (31.7)
Provided feedback to their subordinates on their tasks	0 (0.0)	2 (3.3)	12 (20.0)	22 (36.7)	24 (40.0)
Received constructive feedback from colleagues or subordinates	0 (0.0)	2 (3.3)	19 (31.7)	25 (41.7)	14 (23.3)
Delegated tasks to their subordinates	0 (0.0)	1 (1.7)	9 (15.0)	23 (38.3)	27 (45.0)
Promoted good working relationships among their subordinates	0 (0.0)	0 (0.0)	4 (6.7)	24 (40.0)	32 (53.3)
Built trust with their subordinates	0 (0.0)	0 (0.0)	8 (13.3)	30 (50.0)	22 (36.7)
Practiced and valued shared decision making	0 (0.0)	1 (1.7)	6 (10.0)	31 (51.7)	22 (36.7)
Engaged in team building sessions with members	1 (1.7)	6 (10.0)	10 (16.7)	21 (35.0)	22 (36.7)
Facilitated conflict and alternative dispute resolution among members	10 (16.7)	11 (18.3)	10 (16.7)	17 (28.3)	12 (20.0)
Encouraged a high level of commitment to the purpose and values of the health facility	0 (0.0)	1 (1.7)	12 (20.0)	27 (45.0)	20 (33.3)
Held self and others accountable for health facility organisational goal attainment	0 (0.0)	1 (1.7)	10 (16.7)	21 (35.0)	28 (46.7)
Promoted and managed change within the health facility	1 (1.7)	3 (5.0)	5 (8.3)	32 (53.3)	19 (31.7)
Anticipated and planned strategies for overcoming obstacles at the health facility	1 (1.7)	1 (1.7)	16 (26.7)	28 (46.7)	14 (23.3)
Anticipated the need for resources to carry out initiatives	1 (1.7)	3 (5.0)	8 (13.3)	31 (51.7)	17 (28.3)
Advocated and participated in healthcare policy initiatives	7 (11.7)	8 (13.3)	12 (20.0)	25 (41.7)	8 (13.3)
Supported and mentored high-potential talent within the health facility	4 (6.7)	9 (15.0)	9 (15.0)	26 (43.3)	12 (20.0)
Analysed and evaluated information to support a decision or recommendation	2 (3.3)	3 (5.0)	8 (13.3)	29 (48.3)	18 (30.0)

## Discussion

This study assessed the leadership and management competencies of health managers in Wakiso district, Uganda. From the findings, health managers generally demonstrated low leadership and management competencies. With several studies linking management competencies to individual work performance [33–35], our findings highlight the critical need for addressing these essential aspects of health service delivery. Notably, cognitive skills and self-management dimensions emerged as the strongest in leadership and management competencies respectively. Health managers identified monitoring, speaking and active listening as the most important cognitive skills, while coordination, instructing, and social perceptiveness were prioritised among interpersonal skills. Additionally, health managers frequently engaged in promoting good working relationships among subordinates, holding themselves and others accountable for health facility organisational goal attainment, and creating effective work environments for subordinates. Effective leadership and management competencies have been reported to improve performance in healthcare settings, especially those in low resource settings such as Uganda [1–3]. Strengthening leadership and management competencies among health managers would therefore lead to an efficient healthcare workforce, hence improved healthcare outcomes.

From our findings, only 28.3% and 38.3% of the health managers were inducted and motivated to perform their duties respectively. This finding concurs with previous findings among health managers in three African countries of Ghana, Ethiopia and Tanzania that found that most of the health workers in leadership and management roles did not know what was expected of them [36]. Induction, training and continuous development of workers are critical as these not only enhance workers’ performance in their current jobs but also prepare them for future responsibilities [37]. In addition, induction programmes have been reported to equip managers with the necessary knowledge and skills, enabling them to adapt quickly to their roles and make informed decisions [38]. Studies in Uganda and similar settings have suggested that early exposure to leadership principles during medical education can produce a generation of health workers well equipped to address healthcare challenges [19]. Integrating leadership and management training into health curricula is therefore essential for providing health professionals with ideal competencies during the early stages of their career in the Ugandan context. Motivation among health managers is equally important for achieving optimal healthcare outcomes [39]. Interventions that enhance motivation may include timely payment of salaries, availability of essential resources and supplies, recognition of achievements, support supervision, and opportunities for career development [39,40]. However, these factors are known to affect the health system in Uganda hence impacting the motivation and performance of health managers. Literature suggests that individual’s state of motivation, including job satisfaction, significantly influences their ability to develop as effective leaders [41]. Conversely, demotivation can hinder health managers from exhibiting the leadership behaviours necessary for effective healthcare delivery [42]. This can result in reduced staff morale, lower utilisation of healthcare services, and suboptimal patient outcomes. The Ministry of Health and local governments should therefore prioritise strategies to enhance health managers’ motivation and satisfaction.

The low leadership (25.0%) and management (33.3%) competencies established in our study has been previously reported in several studies in LMICs where majority of health manager appointments into managerial roles is often based on technical and clinical expertise rather than prior leadership and management experience [2,24,43]. In some of these countries, managerial appointments are influenced by factors such as corruption, bribery and political interests, overshadowing the merit based selection criteria [24]. Similarly, in Uganda, health managers frequently assume managerial roles with no specific descriptions of their roles or operational guidelines from the Ministry of Public Service or Local Government Service Commissions [7,44]. In addition, a recent study in Uganda reported little or no exposure of health managers to leadership and management skills and principles before taking up managerial roles, and hardly any university or higher institution of learning was found to provide leadership and management as a course [24]. This implies that most health managers often rely on informal mentorship or ad hoc instructions to carry out their responsibilities, further emphasising the critical need for structured leadership and management guidelines and capacity building programmes in the country.

Our study established a relatively high performance in cognitive (mean = 37) and self-management (mean = 36.5) skills under leadership and management assessments respectively. This may indicate that these competencies are more universally transferable and can be developed through individual efforts and general experience. In addition, health managers perceived monitoring (65.0%), speaking (60.0%) and active listening (58.3%) as the most important cognitive skills, reflecting a positive attitude towards effective leadership and management. Monitoring, a key component of leadership and management, enables managers to assess the performance of their subordinates, health facilities, as well as themselves [31]. Speaking and active listening on the other hand are essential for effective communication. Speaking ensures that managers clearly convey messages, while active listening involves giving full attention to subordinates’ and patients’ complaints as well as taking time to understand, engage and effectively respond to them [29]. For interpersonal skills, coordination (45.0%), instructing (41.7%), and social perceptiveness (41.7%) were mentioned as the most important. Coordination, which involves adjusting actions in relation to those of others, is crucial in team building, while instructing ensures that managers provide clear guidance and training to subordinates, creating a culture of continuous learning and professional development [29,31]. Social perceptiveness, which entails being aware of and understanding others’ reactions, helps managers to understand staff’s and patients’ emotions, needs and concerns for mitigation of conflicts and enhancement of teamwork [45]. These skills are fundamental in creating a strong work environment that promotes efficiency and positive patient outcomes. Furthermore, financial (63.3%), logistic (56.7%) and human resources (52.5%) were the three critical business analysis skills. Effective financial management ensures the optimal use of funds, preventing wastage, and enabling investments in key areas of the health facility such as infrastructure and staff welfare [46]. Logistic resource management, which involves ensuring the availability and distribution of medical supplies and equipment, reduces on the chances of the health facility running out of essential medical supplies and drugs, while human resource management maintains staff motivation [47]. Together, these skills lead to effective healthcare management, ensuring that resources are utilised efficiently to meet the needs of both health facility staff and patients.

Health managers in our study reported most frequently engaging in promoting good working relationships among subordinates (53.3%), holding self and others accountable for health facility organisational goal attainment (46.7%), and creating an effective work environment for subordinates (45.0%). These practices are promising as they contribute to creating a collaborative and motivated workforce which is essential for delivering quality and effective healthcare services. Evidence suggests that good working relationships between health workers and health managers results in doing a good job and is a means for creating belongingness at work [48]. This therefore builds trust and teamwork among staff as they remain focused on achieving the health facility goals. A healthy work environment includes effective leadership, communication, teamwork and professional autonomy [49]. This enhances staff morale and reduces burnout among staff, thereby ensuring effective healthcare service delivery which is crucial in the Ugandan context. However, advocating and participating in healthcare policy initiatives (20.0%), facilitating conflict and alternative dispute resolution among members (20.0%), and supporting and mentoring high-potential talent within the health facility (20.0%) were the least practiced activities. The finding of limited participation in advocacy and policy initiatives corroborates with previous studies [50,51] which reported advocacy limiting factors such as low formal training in advocacy and policies, heavy workload, and limited guidance on engaging in policy and advocacy activities. In the local context, health advocacy is crucial for influencing policy decisions that improve healthcare service delivery and close health disparity gaps. Conflict resolution skills are vital for maintaining harmony and preventing disputes from subordinates, while mentoring high-potential staff is important for building competent future leaders as well as ensuring the continuity of strong leadership within health facilities. Addressing these gaps by district authorities and Ministry of Health requires targeted interventions, such as training programmes that emphasise advocacy skills, conflict management, and mentorship. In addition, creating platforms for health managers to participate in policy discussions and contribute to decision-making processes can empower them to advocate for necessary reforms effectively.

Our study had some limitations which may need to be considered during interpretation and use of the findings. District health managers and top officials from the Ministry of Health were not involved in the study which may have excluded their potentially valuable perspectives on systemic challenges and solutions. The study was limited to 60 health managers from health facilities directly supervised by Wakiso District Local Government, which represents a small sample size of local government health managers and reduces the generalisability of the findings nationally. Indeed, findings from the study may not be relatable to other districts in the country that have different socio-demographic characteristics from Wakiso. While the study cut-off points used for measuring leadership and management competencies were established through consultation with government officials and the research team, we acknowledge that this approach has not been empirically examined. However, given the absence of standardized methodologies for determining cut-off points in our study context, this approach was considered the most appropriate method to provide valuable insights and ensure alignment of results with current national public service standards. Furthermore, the reliance on self-reported information introduces the potential for social desirability bias. Despite these limitations, the study has several notable strengths. It is one of the few studies that have focused on leadership and management competencies among lower-level health facility managers in Uganda. Previous studies predominantly concentrated on district-level managers and administrators, leaving a gap in understanding leadership and management in the lower-level facilities of the health system. In addition, the inclusion of health facilities in urban, peri-urban, and rural settings in Wakiso district ensured that the study captured diverse perspectives that reflected the varied contexts within the district. Future research on leadership and management may engage policy makers at national level and other stakeholders such as implementing partners, as well as involve districts from different regions of the country.

## Conclusions

This study highlights the competency gaps in leadership and management of health managers in Wakiso District, Uganda, as well as promising practices for enhancing healthcare service delivery. Most performed practices such as promoting good working relationships, holding everyone accountable for the achievement of health facility goals, and creating conducive work environments can positively influence service delivery. However, limited engagement in activities such as advocacy, conflict resolution, and mentorship may limit the provision of good healthcare services. These findings underscore the urgent need for addressing the leadership and management challenges among health managers through continuous training, supervision, and mentorship for effective health service delivery in Wakiso District and similar settings.

## Supporting information

S1 Dataset This is the study dataset. (XLSX)

S2 Inclusivity in global research This is the completed questionnaire on inclusivity in global research. (DOCX)
